# Technological Feasibility of Couscous-Algae-Supplemented Formulae: Process Description, Nutritional Properties and In Vitro Digestibility

**DOI:** 10.3390/foods10123159

**Published:** 2021-12-20

**Authors:** Sheyma Khemiri, Maria Cristiana Nunes, Rui J. B. Bessa, Susana P. Alves, Issam Smaali, Anabela Raymundo

**Affiliations:** 1 Laboratory of Protein Engineering and Bioactive Molecules (LR11ES24), National Institute of Applied Science and Technology, University of Carthage, BP 676, Tunis 1080, Tunisia; khemirisheyma@gmail.com (S.K.); Issam.smaali@insat.rnu.tn (I.S.); 2 LEAF—Linking Landscape, Environment, Agriculture and Food, Instituto Superior de Agronomia, Universidade de Lisboa, Tapada da Ajuda, 1349-017 Lisboa, Portugal; crnunes@gmail.com; 3 CIISA, Faculdade de Medicina Veterinária, Avenida da Universidade Técnica, Pólo Universitário do Alto da Ajuda, 1300-477 Lisboa, Portugal; rjbbessa@fmv.ulisboa.pt (R.J.B.B.); susanaalves@fmv.ulisboa.pt (S.P.A.)

**Keywords:** couscous-algae, nutritional properties, rheological properties, in vitro digestibility, sensory evaluation

## Abstract

The aim of this work was to develop functional couscous in a traditional Tunisian manner (hand rolling), enriched in algae biomass (6% *w/w*). Four *Chlorella vulgaris* (*C. vulgaris*) biomasses and one mixture of *C. vulgaris* and two macroalgae biomasses (*Ulva rigida* and *Fucus vesiculosus*) were used. The *C. vulgaris* strain was subjected to random mutagenesis and different culture conditions (Allmicroalgae), resulting in different pigmentations and biochemical compositions. Couscous samples were characterized in terms of nutritional properties, oscillatory rheology properties and digestibility. All biomasses provided a significant supplementation of nutrients and excellent acceptance. The enrichment resulted in lower firmness, higher viscoelastic functions (G′ and G″) and a significant improvement in the cooking quality. Major differences between couscous samples with different microalgae were observed in protein and mineral contents, fully meeting Regulation (EC) No. 1924/2006 requirements for health claims made on foodstuffs. The amount of digested proteins was also higher in algae-containing samples. The fatty acid profile of the enriched couscous varied in a biomass-specific way, with a marked increase in linolenic acid (18:3 ω3) and a decrease in the ω6/ω3 ratio. Sensory analysis revealed that microalgae-containing products could compete with conventional goods with an added advantage, that is, having an ameliorated nutritional value using algae as a “trendy” and sustainable ingredient.

## 1. Introduction

The link between diet and health is currently growing very steeply. People are becoming used to the consumption of sophisticated and innovative products. This is caused by many factors such as increased health care costs and population aging, thus increasing consumers’ interest in attaining a state of well-being through diet. The new way of conceiving foods originated the so-called functional products and induced a higher consumer demand for these types of goods. Currently, greater focus is being placed on sustainable “green” sources of food that produce compounds with efficient biological activity, which could be used as functional ingredients or dietary supplements. These biomolecules include polyunsaturated fatty acids (PUFAs), proteins, vitamins and minerals [1,2].

Algal biomass and products thereof figure at the top of the list in the food market as sustainable nutritional bio-ingredients and are promoted as “superfoods” which could be used in the formulation of “trendy” goods [3]. Consumption of algae began in the ninth century in Africa using *Arthrospira* (a cyanobacteria commonly known as *Spirulina*) as food [4]. In recent years, an important number of scientific works have evaluated the potential of algae biomass to be used as a functional ingredient in baked goods [5,6,7], pasta [8], milkshakes, vegetable soups and snacks [9], and some products are currently commercially available such as Züpa Superfood Soup (Zupa Noma, Santa Clara, CA, USA) and Chia & Spirulina Roo’Bar (Roo Brands, Sofia City, Bulgaria). Despite the increasing effort from researchers and food technologists to add value to algae biomass, its use for the enrichment of food products is still limited to an industrial scale [10]. This mainly results from the regulation provisions, as only a limited number of species are currently authorized by the European Food Safety Authority (EFSA) for human consumption [11]. Furthermore, the reluctance of consumers to accept the products, due to the green color and the strange aroma, flavor/smell and taste, may be considered another limiting factor for upscaling.

Algae biomasses comprise a wide spectrum of bioactive compounds which vary mainly depending on the strain and the culture conditions, such as the physiological status and growth phase [12]. The difference in the algae’s biochemical profile may impact the physical and sensorial properties of the biomass [13], and consequently those of algae-containing products. 

The organoleptic characteristics of algae biomass are strongly linked to the consumers’ acceptance since color is the first parameter taken into consideration by consumers and can be decisive for the inclusion of the food in their diet [9]. Therefore, consumers usually characterize green-colored algae-based food products with a low sensorial profile. Alternative strategies to boost the organoleptic characteristics (appearance and taste) of foods containing algal biomass could help make them more acceptable to consumers [8]. Examples include the addition of other natural ingredients to improve the flavor and modify the food color, the combination of hetero- and photoautotrophic growth of wild types of microalgae (only possible for some strains) [14] and chlorophyl-deficient mutants obtained by chemical-induced mutagenesis [15]. Another approach is the extraction of the microalgae target compounds with the concomitant removal of chlorophyll [16].

The aim of the current work was to develop a novel food product with a high concentration (6%) of *Chlorella vulgaris* (*C. vulgaris*) biomass with different pigmentations. A mixture of microalgae (*C. vulgaris*) and seaweeds (*Fucus vesiculosus* and *Ulva rigida*) was also used. It was predetermined to use an elevated concentration of algal biomass compared to the concentrations typically present in commercial products (<1%) in order to bring higher levels of bioactive molecules that are sufficient to functionalize the product. The food matrix in which the algae biomass was incorporated was couscous, an ethnic staple food product of some of the Maghreb countries or the Berber world (Northern Africa). This cereal product is known for its simplicity in terms of the raw materials used and the cooking process, in addition to its nutritional characteristics such as its low fat content and the presence of some vitamins and minerals [17]. A few scientific publications are available concerning the original food product [18,19]; some have evaluated the differences between its industrial and typical versions [20]. Likewise, others have evaluated the effects of the use of unconventional flours on nutritional and sensorial properties [19]. Some industries have started to produce couscous worldwide; nevertheless, replicating the quality of the traditional home-made product remains a challenge. It is worth noting that the traditional steps followed are quite complex, which makes the ethnic products less consumed in favor of the industrial ones. This may be justified by globalization in addition to economic concerns [17].

The current paper discusses the overall quality and the acceptability of the end product. The variables evaluated include color, texture and mechanical features. In vitro gastrointestinal digestion was performed to mimic physiological conditions to evaluate the degree of nutrient bioaccessibility. A sensory analysis that included assessment of flavor, overall acceptance and purchase intention was conducted.

## 2. Materials and Methods

### 2.1. Microalgae and Other Ingredients

*Chlorella vulgaris* AGF002 (*C. vulgaris*) and Algaessence^®^ biomass were provided by Allmicroalgae Natural Products (Pataias, Portugal). *C. vulgaris* was cultivated in auto- and heterotrophic modes. Guillard’s F2 medium [21] was adjusted and used as an autotrophic medium. The heterotrophic culture was created in a bench-top fermenter in semi-continuous mode (New Brunswick BioFlo^®^ CelliGen^®^115; Eppendorf AG, Hamburg, Germany), using glucose as the organic carbon source with a C: N ratio of 6.7:1. Chemically random mutagenesis was induced in order to develop chlorophyll-deficient *C. vulgaris* mutants with different pigmentations [15]. The biomass was collected aseptically, centrifuged (VWR Micro Star 12, Radnor, PA, USA), spray dried, powdered and stored, until analysis and use. The obtained biomasses were confirmed as autotrophic *C. vulgaris* (Organic *Chlorella*) and heterotrophic *C. vulgaris*: Smooth *Chlorella* (light green color), Honey *Chlorella* (yellow color) and White *Chlorella (white color*). Algaessence^®^ is a micro- and macroalgae blend composed of Organic *Chlorella, Fucus vesiculosus* and *Ulva rigida*. Macroalgae biomasses were cultivated by ALGAplus (Ílhavo, Portugal), and the mixture was packaged and commercialized by Allmicroalgae Natural Products. Except for the mineral profile and chlorophyll content, the chemical composition of the different algae biomasses used was provided by Allmicroalgae as indicated in Table 1. The main differences in composition are highlighted in bold.

Medium semolina (100% durum wheat) from “Pâtes Warda” (Sousse, Tunisia) was used for couscous production (Table 1).

### 2.2. Couscous Preparation

**Figure 1:**
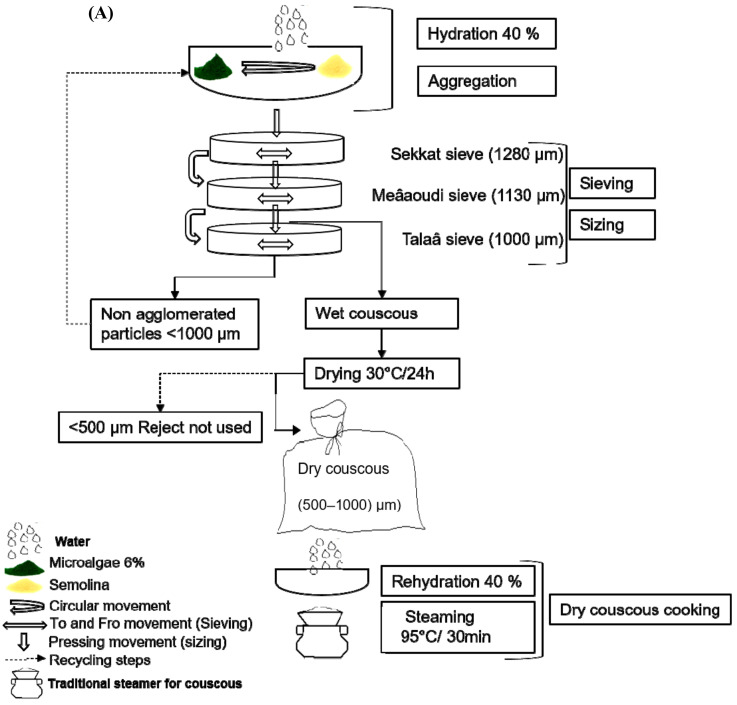

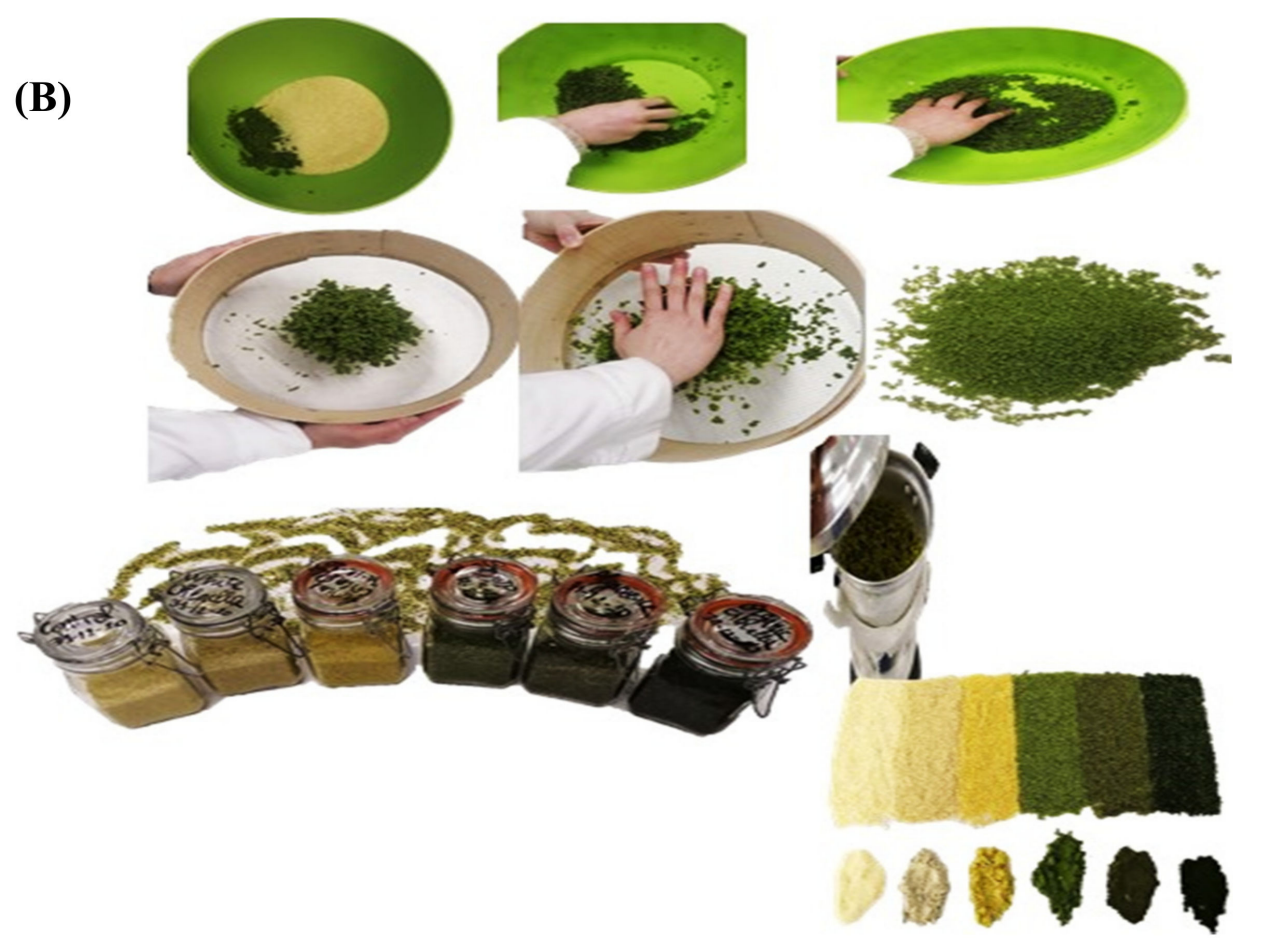
Schematic representation (**A**) and photographs (**B**) of couscous manufacturing/cooking according to the traditional Tunisian procedure.

Couscous was produced employing an optimized traditional Tunisian handmade procedure, and all the steps are summarized in Figure 1. Durum wheat semolina was used at 100% to make the control sample, and 6% (*wt*/*wt*) of semolina was replaced by the different types of algae. The flour was first moistened with water (40%) in a dish, followed by circular movements to ensure an adequate homogenization. The moistened semolina then underwent the sizing and sieving steps, using three sieves with mesh openings decreasing from 1280 to 1000 µm. Sizing aims to force all the particles through the holes of the sieve, by moving and pressing them with the palm of the hand. No agglomerated couscous particles (<1000 µm) were recycled, and rolled couscous grains up to 1000 µm were dried at 30 °C/24 h (Arianna XLT133, Unox, Cadoneghe, Italy). The granulometry of the dried couscous was controlled by sieving on 500 µm and 1130 µm sieves in order to eliminate fine and large particles. Three batches of 100 g of couscous dough were developed for each formulation. The dry couscous was rehydrated (40% of water) and rolling movements were applied for 10 min, in order to help the grains absorb the water sufficiently. The hydrated granules were placed in the superior section of a couscoussiere (steaming pot), while the lower section contained boiling water. The steaming lasted 30 min. The steam cooking procedure is considered the last step and was repeated twice. The steam-cooked couscous was set to cool down and then stored at −20 °C.

### 2.3. Cooking Quality Assessment

The cooking quality variables of the couscous samples were determined in terms of cooking loss (CL), swelling, water absorption index (WAI) and water solubility index (WSI). The swelling was determined as described by Ounane et al. [22], by mixing 20 g of uncooked couscous with 50 mL of boiling water in a graduated 100 mL test tube. After ten rotation movements, another 50 mL of boiled water was added, the test tubes were placed in a controlled water bath (25 °C) and the couscous volume was registered after 30 min. Drained cooking water was dried to a constant weight and the extent of CL was calculated. For WAI, 2.5 g of couscous was mixed with 30 mL of water, shaken for 30 min and then centrifuged at 2200 g for 10 min. The pellets were weighed and the WAI was calculated [17]. The obtained supernatant was drained carefully into a pre-weighed container, placed at 105 °C and then weighed on an analytical balance. The WSI was calculated as the ratio between the weight of the dried supernatant and the dry weight of the sample [17]. Cooking quality determinations were repeated at least in triplicate.

### 2.4. Color Analysis

The color of uncooked and steam-cooked samples was instrumentally (CR400 Chromameter, Minolta, Japan) evaluated according to CIELAB system color parameters (L*, a* and b*), where L* indicates brightness (values increase from 0 to 100), a* indicates the degree of redness or greenness (+60, red; −60, green) and b* indicates the degree of yellowness or blueness (+60, yellowness; −60, blueness). The measurements were conducted under the same artificial fluorescent light using a white standard (L* = 94.61, a* = −0.53, b* = 3.62), and the same temperature (20 ± 1 °C). The measurement was replicated at least eight times.

### 2.5. Rheology Characterization Plate System

The small amplitude oscillatory shear (SAOS) behavior of the couscous dough (before sieving) was measured in a controlled-stress rheometer (Haake Mars III—Thermo Scientific, Karlsruhe, Germany) with a UTC-Peltier system. The sample was placed on the bottom of a serrated parallel-plate sensor with a 35 mm diameter (PP35) and a 3 mm gap (previously optimized). After placing the rheometer in the measuring position, with a 3 mm gap between plates, the edges were coated with liquid paraffin to prevent moisture losses during tests. The linear viscoelastic region (LVER) was previously accessed for all the samples, at 6.28 rad/s (1 Hz), through a stress sweep test, and in all the samples, a constant shear stress of 20 Pa was applied to perform the frequency sweep tests (from 0.0628 to 628 rad/s). All rheology measurements were repeated at least three times. Storage moduli (elastic) G′ and loss moduli (viscous) G″ (Pa) data versus angular frequency ω (rad/s) were fitted through power equations, where α′, α″, b′ and b″ are the corresponding fitting parameters (Equations (1) and (2)) [23].

G′ (ω) = α′ ω^b′^ (1)

G″ (ω) = α″ ω^b″^ (2)

### 2.6. Texture Analysis

The couscous texture was evaluated through a texturometer, TA-XTplus (Stable MicroSystems, Surrey, UK), in compression mode (50%) using a cylindrical aluminum probe with a diameter of 30 mm and 9 mm of penetration distance at 1 mm·s^−1^ crosshead speed. Before each test, the couscous grains were cooked as described above and left to cool down to 20 °C for 1 h. The steam-cooked couscous (20 g) was placed in cylindrical glass cases (4 cm diameter and 4.2 cm height) and measurements were repeated at least six times for each sample. Resistance to penetration was expressed as firmness (N) and corresponds to the maximum force value.

### 2.7. Proximate Chemical Composition and Fatty Acid Determination

Moisture and ash content were determined conforming to ICC methods 110/1 and 104/1, respectively. The total fat content was measured by acidic hydrolysis, followed by centrifugation and extraction with n-hexane. The crude fat residue was determined gravimetrically, after rotary evaporation and N_2_ gas. The mineral profile was evaluated by inductively coupled plasma optical emission spectrometry (ICP-AES: Thermo System, ICAP-7000 series) [24]. The crude protein content was measured by Dumas (Thermo Quest NA 2100 Nitrogen and Protein Analyser, Interscience, Breda, the Netherlands), using a protein-to-nitrogen conversion factor of 6.25. The carbohydrate content was calculated as the difference between the protein, lipid, ash and moisture contents.

Concerning pigment determination, aliquots of algal extracts were prepared at 1 mg/mL (in ethanol), absorbances were measured at 470, 648 and 664 nm and then the chlorophyll content was calculated using the Lichtenthaler equations [25]. Fatty acid composition was analyzed by gas chromatography (Shimadzu GC 2010-Plus; Shimadzu, Kyoto, Japan), using a fused-silica capillary column (SP-2560; 100 m, 0.25 mm i.d., 0.2 μm film thickness, Supelco Inc., Bellefonte, PA, USA), equipped with a flame ionization detector (GC-FID) as described by Bessa et al. [26]. The identification of some unknown peaks was determined by gas chromatography-mass spectrometry (GC-MS), using a Shimadzu GC-MS QP2010 Plus (Shimadzu, Kyoto, Japan) equipped with an SP-2560 (100 m, 0.25 mm i.d., 0.2 μm film thickness). Fatty acids were expressed as the percentage of the total fatty acid content. All chemical composition analyses were repeated in triplicate, performed on steam-cooked couscous and expressed in percentage *w/w*, dry basis.

### 2.8. In Vitro Digestion: INFOGEST Static Model

The cooked couscous samples’ in vitro digestibility was evaluated following the INFOGEST protocol [27]. The enzyme activities and bile concentration were measured prior to the digestion experiment as described by Minekus et al. [27]. Each sample was mixed (2:2 *wt*/*wt*) with simulated salivary fluid (SSF) containing amylase (300 U/mL) for 2 min at pH 7. The mixture was then diluted with simulated gastric fluid (SGF) containing gastric enzyme (Pepsine 2000 U/mL) and incubated for 2 h (pH 3). Subsequently, simulated intestinal fluid (SIF) containing pancreatin (100 U/mL) and bile salts (20 mM) was added and incubated for a further 2 h (Ph 7). The whole digestion protocol was performed at 37 °C, under constant gentle mixing on a rotating wheel. Finally, the intestinal phase was stopped using the protease inhibitor 4-(2 aminoethyl) benzensulfonylfluorid (AEBSF, trademark Pefabloc^®^, 500 mmol/L, Roche, Basel, Switzerland). A reagent blank was also prepared. All samples were finally centrifuged (18,000× *g* at 4 °C), and the undigested residues were collected and dried at 80 °C for 6 h, and then at 45 °C, until constant weight.

The dry matter and crude protein in vitro digestibility (%) of all the couscous products was calculated from the difference between the initial biomass and the undigested biomass, divided by the initial biomass and multiplied by 100. Analyses were repeated in triplicate.

### 2.9. Sensory Evaluation

Sensory analysis assay was performed for couscous enriched with Honey, Smooth and Organic *Chlorella* commercial biomasses at a 6% (*wt*/*wt*) incorporation level. The test was conducted in a standardized sensory analysis room as previously described by Khemiri et al. [5]. Samples were prepared and cooked according to the procedure described above and served cooled, 1 h later. An untrained group of 24 females and 9 males, aged between 25 and 50, participated in the sensory sessions. Couscous samples were assessed for the following attributes: color, odor, taste, texture and global appreciation (9 levels from “very pleasant” to “very unpleasant”). Buying intention was also evaluated (from “would certainly buy” to “certainly wouldn’t buy”).

### 2.10. Statistical Analysis

Statistical analysis was performed using Origin Pro 8.0 software (OriginLab Corporation, Northampton, MA, USA), through analysis of variance (one-way ANOVA). When significant effects (*p* < 0.05) of treatments were detected in ANOVA, multiple comparisons of means were conducted using Tukey’s approach. Principle component analysis (PCA) (OriginLab Corporation, Northampton, MA, USA) was carried out to achieve a better understanding of the relationship between the different sensory attributes. All results are reported as average ± standard deviation (SD).

## 3. Results and Discussion

### 3.1. Cooking Quality

**Figure 2:**
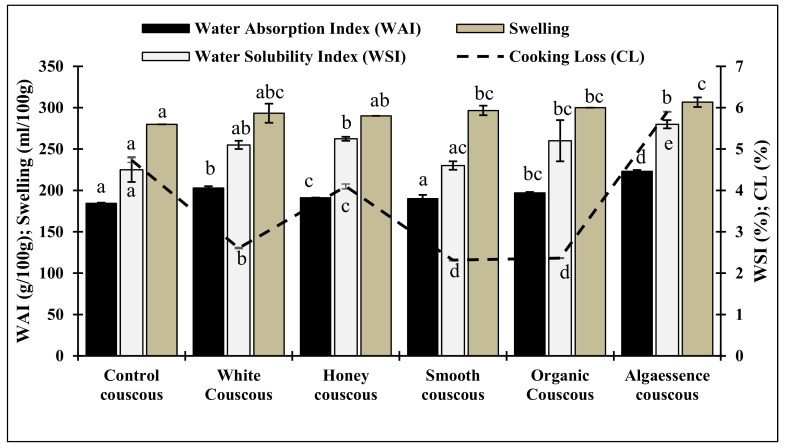
Technological characteristics of couscous with 6% algae supplementation. Control is couscous without algae addition. Data shown are mean ± SD, *n* = 3. Distinct letters in each bar indicate significant differences (*p* < 0.05).

The results obtained for the couscous cooking behavior are presented in Figure 2. Globally, all values obtained for the innovative couscous were either comparable or higher than those detected in the control sample. The ability of couscous to swell and rapidly absorb sauces is considered to be an important criterion for the overall couscous cooking quality by both consumers and industry [17]. The extent of couscous swelling can vary according to the raw materials used [22]. Values of water absorption capacity were the highest in Organic (197 g/100 g), White (203 g/100 g) and Algaessence (223 g/100 g) couscous samples. This resulted from the higher holding capacity of polysaccharides, proteins and fiber in the Organic, White and Algaessence (Table 1) *Chlorella* biomasses, resulting in high-quality products. Accordingly, *Chlorella* biomass incorporation positively affected couscous cooking loss as the microalgae-couscous presented significantly (*p* < 0.05) lower values (Figure 2). Cooking loss is mainly influenced by the dissolution and release of gelatinized starches from the surface of couscous grains into the cooking water. This release could be limited by the lipid presence in microalgae biomass which complexes with amylose during cooking, thus reducing its disintegration [19]. The WSI, which expresses the extent of couscous disintegration during water absorption, ranged between 4 and 6%, indicated by Abecassis et al. [28] as classical values for semolina couscous. Overall, the product developed in the present study can be regarded as a high-quality product.

### 3.2. Color Stability

The presence of algae biomass in the formulation of couscous led to variations in color compared to the control couscous (Table 2), as expected. Regardless of the biomass used, raw couscous samples presented significantly lower lightness, a reduction in yellowness b* (except for the Honey couscous) and an increase in greenness −a* (a*, in modulus). Color is an aspect of appearance that plays a major role in the acceptability of a food product. The darkness obtained after algae addition does not penalize the couscous since consumers are already accustomed to purchasing vegetable-rich products, identifying this feature with darker colored products. Couscous prepared with *Chlorella* (autotrophic and heterotrophic) is visually very attractive, presenting yellow or different shades of green (Figure 1B). The color differences are explained by the difference in pigment content in the used microalgae biomasses (Table 1). The highest greenness (−a*) value was determined in the Organic couscous due to the presence of a high chlorophyll content in the Organic *Chlorella*, followed by the Algaessence and Smooth couscous. The color stability was evaluated, and cooking resulted in differences (*p* < 0.05) within the same color parameter. Color losses resulting from the couscous cooking process are related to pigment oxidation, besides a certain degree of leaching [8,29]. The higher the chlorophyll content, the lower the difference in the colors between raw and cooked samples.

### 3.3. Mechanical Properties

#### 3.3.1. Rheological Properties of Couscous Dough

**Figure 3:**
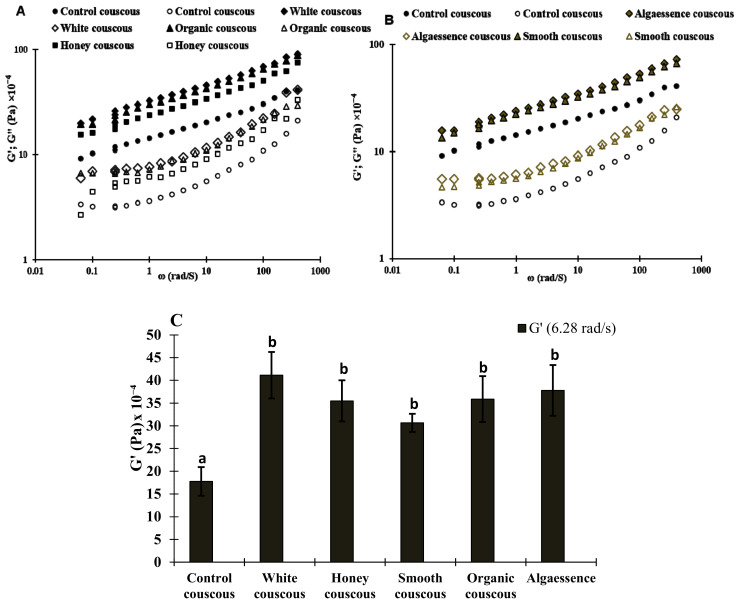
Mechanical spectra (**A**,**B**) of couscous dough with algae biomass and values of G′ at 6.283 rad/s (1 Hz) (**C**). Control is couscous dough without algae addition. Closed symbols—G′; open symbols—G″. Different letters indicate that the difference in the means is significant at the 0.05 level.

The results from the small amplitude oscillatory shear (SAOS) measurements of the uncooked couscous are expressed in terms of storage (G′) and loss (G″) moduli (Figure 3). All the samples presented a viscoelastic behavior characterized by G′ > G″, and both moduli depended on the frequency. Compared to the control couscous dough, the incorporation of 6% algae caused a significant reinforcement of the dough structure. This can be confirmed by comparing the G′ values obtained at 6.283 rad/s (Figure 3C). The frequency dependence of G′ and G″ could be described by the power law equations (Table 3). As indicated in Table 3, the replacement of 6% semolina by algae biomass increased α′ values. The restructuring effect is more visible in the couscous with White and Organic *Chlorella* with the highest protein contents (Table 1). It appears that protein from *Chlorella* took the head in the structure network. Studies relating to the impact of algae in couscous dough rheology have not been published to date. However, Nunes et al. [6] studied the impact of microalgae cell disruption pretreatment on the dough rheology and found that 1% *Chlorella vulgaris* had a negative impact on the dynamic viscoelastic properties of the wheat bread dough. From another viewpoint, the addition of curd cheese and fresh yoghurt promoted the reinforcement of the wheat bread dough structure, expressed in terms of viscoelastic function (G′ and G″) increases [24]. It is worth noting that durum wheat is rich in gluten, but that it is not readily available as the starchy endosperm is hard to break to release the gluten network, which expresses its quality after applying mixing techniques. This feature makes the uncooked dough split easily, and thus it is easier to shape (sieving and sizing) to make the couscous grains, for instance. The rigid dough obtained after algae addition can be corrected by the adjustment of the added water to achieve the desired softness of the dough and thus ease the sieving process.

#### 3.3.2. Texture of Steam-Cooked Couscous

**Figure 4:**
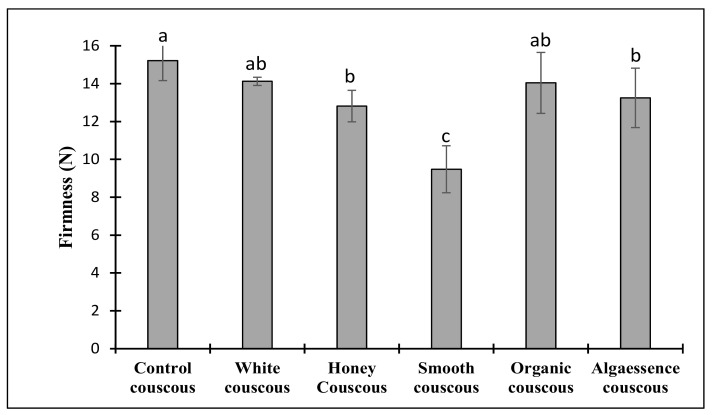
Firmness of cooked couscous with algae incorporation. Control is couscous without algae addition. Data shown are mean ± SD, *n* = 6. Different letters in each bar indicate significant differences (*p* < 0.05).

After steam cooking, the couscous texture properties were assessed by a penetration test. The main mechanical properties of the cooked couscous quality included lower firmness and stickiness. The results of couscous firmness are presented in Figure 4. The addition of algae resulted in a significant (*p* < 0.05) decrease in the cooked couscous firmness (9–14 N) in comparison with the control sample (16 N). The difference in firmness mainly arose from the difference in the gluten fraction. An increase in the gluten content results in a structural reinforcement and vice versa. In the current investigation, decreasing the gluten amount by replacing semolina with algae biomass resulted in structure weakening. The decrease in the firmness was significantly noticeable in couscous with Smooth and Honey *Chlorella* with the lowest protein content (Table 1). The firmness of steam-cooked couscous is probably related to starch–protein complex formation. This suggests that during steam cooking, proteins absorb water, engendering the concentration of the dispersed phase to rise, thus providing a mechanical support for the starch. It seems that proteins from microalgae biomass duplicate the role of the native proteins in wheat semolina. It was reported that a protease treatment, causing a protein disruption, resulted in a decrease in the firmness of cooked rice. The authors related this to the reduction in the protein’s intermolecular interactions due to enzyme treatment [30]. The lipid fraction is also thought to contribute to the hardness of food matrixes [31]. This is probably due to lipid–amylose complex formation [30].

Additionally, the softness obtained in the Algaessence couscous could be due to the high fiber content in the Algaessence biomass (Table 1). Fiber disrupts the protein–starch matrix within the couscous microstructure, thus resulting in a firmness reduction [32]. The rheology data do not corroborate these findings, but we compared two different materials (dough and cooked couscous). The real reinforcement of the structure is only visible after heat treatment when starch gelatinization occurs. Although the effect of the addition of algae to couscous, for the tested level, was smoothened by the cooking process, it was independent of the alga source. Nevertheless, there are no significant differences (*p* < 0.05) between the Control, White and Organic couscous firmness values. 

### 3.4. Nutritional Profile

Innovative couscous samples were prepared by replacing 6% (*wt*/*wt*) of semolina with algae biomass. As predicted, the chosen ratio permitted product functionality. Couscous samples showed varying chemical compositions depending on the types of algae used (Table 4). All samples presented high moisture values coherent with the results from water absorption capacity (Figure 2). Couscous with incorporation of algae biomass showed a significantly lower carbohydrate content and higher ash levels than the control (46.39 g/100 g and 0.71 g/100 g, respectively). The incorporation of *Chlorella* biomass resulted in substantial improvements in the protein content in the final product due to the high protein content of microalgae biomass (Table 4). Differences in the protein content of the five biomasses used in this study resulted in differences in the protein content for the algae-enriched couscous (Table 1). The Organic couscous presented the highest protein content (7.51 g/100 g). Proteins provide at least 12% of the energy value of the enriched couscous samples, which means they can potentially use claims in relation to providing protein according to Regulation (EC) No. 1924/2006. Additionally, the lipid content increased significantly with algae addition, always being lower than 3%. Overall, couscous prepared with algae incorporation presented energy values lower (*p* < 0.05) than the control sample (223.11 kcal/100 g), with advantages in terms of nutritional performance. As far as the mineral content is concerned, a significant improvement in major and trace minerals was determined (Table 4). This improvement was due to the richness of the algae biomass in essential elements (Table 1), representing, in some cases, more than 15% of the recommended daily values (Regulation (EC) No. 1924/2006; Directive No. 90/494 (EC)). These values are highlighted in bold in Table 4. According to the regulation mentioned above, the amount of iron found in functional couscous allows the assertion of the nutrition claim “High in iron” as the Organic and Algaessence couscous contain at least twice the value of 15% RDV (6.82 and 5.74 mg/100 g, respectively). Additionally, the “Source of magnesium” claim can be made due to the interesting magnesium content found in the Algaessence couscous sample (64.20 mg/100 g). Accordingly, health claims such as “microalgae-couscous contains iron which contributes to the normal function of the immune system” and “magnesium contributes to a reduction in tiredness” could be stated.

The fatty acid profile (percent of total fatty acids) of steam-cooked couscous is presented in Table 5. Polyunsaturated fatty acids (PUFAs) were the dominant group, representing more than 50% of the total fatty acids in all samples. Among PUFAs, linoleic acid (18:2 ω6) was the major fatty acid in the couscous, representing around 65% (Organic couscous) to 77% (control couscous) of total PUFAs. Palmitic acid (16:0) was the main saturated fatty acid (SFA) while oleic acid (18:1 ω9) was the most important MUFA detected in both control and algae-containing couscous. The predominance (over 50%) of 18:2 ω6 in grain cereals explains the imbalanced ω3/ω6 present in most human diets [33]. Generally, a healthy ratio of omega-ω6 to omega-ω3 appears to be between 2:1 and 4:1 [34]. Incorporation of algae led to increased levels of ω3 fatty acids such as eicosapentaenoic acid (C20:5 ω3) and α-linolenic (C18:3 ω3), finally bringing down the ratio of ω6/ω3 from 18:1 (control couscous) to 8:1 and 5:1 in the Algaessence and Organic couscous, respectively. This has also been observed for algae-enriched semolina pastas reported by other authors [33,35].

The addition of *Chlorella* and Algaessence*^®^* biomass to couscous may be an appealing way to increase the daily intake of functional nutrients, such as minerals and polyunsaturated fatty acids. This supplementation would promote and prevent different types of disorders such as gastric ulcers, constipation, anemia, hypertension, diabetes, infant malnutrition and neurosis [36,37].

### 3.5. In Vitro Digestibility

**Figure 5:**
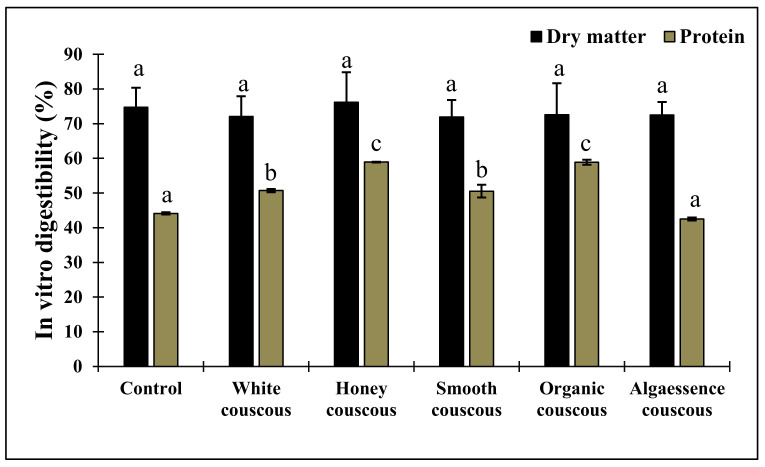
In vitro digestibility (%) of couscous supplemented with algae. Control is couscous without algae addition. Data shown are mean ± SD, *n* = 3. Different letters in each bar indicate significant differences (*p* < 0.05).

Digestibility is an important factor in determining the level of nutritive factors. Thus, if algae are to be used as functional ingredients, it is crucial to know their impact on digestibility. Only few papers have studied the in vitro digestibility (IVD) of algae-based foods [8,38]. To our knowledge, no studies are available regarding the in vitro digestibility of algae-based couscous. In the present study, no significant difference in the dry matter IVD between algae containing couscous at 6% addition and the control was found (Figure 5). In a previous study [8], the addition of 3% *Arthrospira platensis* to gluten-free pasta corresponded to a significant decrease in digestibility. Although the dry matter IVD was similar for all couscous samples, differences emerged in protein digestibility. Couscous supplemented with Honey and Organic *Chlorella* presented a significant increase in IVD, while couscous with Algaessence^®^ biomass was similar to the control sample. The in vitro digestibility of microalgae-based foods may vary according to the species, cell wall structure and nutritional composition [8].

### 3.6. Sensory Evaluation

Sensory analysis of enriched couscous highly contributes to its potential future commercialization, since it provides a perspective of the potential product’s acceptability in the market. Couscous with microalgae biomass presented an attractive and innovative appearance, as seen in Figure 1B. Sensory analysis was performed on cooked couscous with the Honey, Smooth and Organic *Chlorella* biomasses. The selection of the samples was based on protein digestibility results, and also on gaining a better assessment of the impact of pigment colors in the sensory assays. Khemiri at al. [5,39] and Nunes et al. [6] recently reported high visual acceptability scores of a green gluten-free bread, ricotta and wheat bread, respectively, formulated with microalgae biomass. To our knowledge, no studies have reported the sensory analysis of microalgae-containing couscous.

PCA was carried out to evaluate the efficiency of the nine-point hedonic scale on the classification of the different sensory attributes of the tested samples and to better understand the relationship between the attributes within the same sample. 

The sensory attributes of the Honey and Smooth couscous samples are positioned on the right side of the score plot, while the Organic sample attributes are grouped on the left. The loading plot (biplot) shows that the first principal component, which mainly separates the Honey and Smooth samples from the Organic couscous, is dominated by the highest “like” levels on the hedonic scale (like very much and like extremely) on one side and the highest “dislike” levels (dislike slightly, moderately and extremely) on the other side. This means that the first component of the data, accounting for 36% of the total variability, was effective in separating the samples from each other based on the extreme degree of preference. The texture and color of the Organic sample are better described by the second principal component, which explains 21.74% of the total variability. Additionally, sensory attributes of the samples with the same degree of preference are clustered together. Some sensory attributes, more than others, contribute to the overall acceptability of the product. In the case of the Honey and Smooth couscous, the flavor and the color were the most influential attributes for global appreciation since they have close scores along PC1. As far as the Organic couscous is concerned, the global appreciation of the sample is related to the flavor and odor. The flavor and the odor of the Organic couscous were least appreciated and were highly correlated on the basis of PC1. 

**Figure 6:**
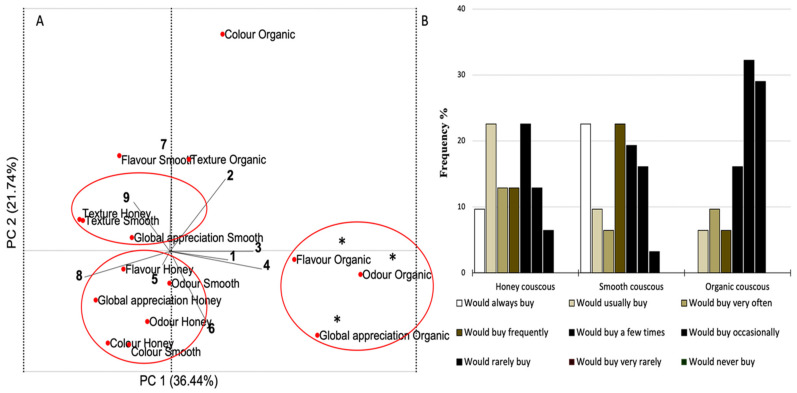
Responses of the panelists (9: extremely like; 1: extremely dislike) regarding sensory attributes (**A**) and buying intention (**B**) (*n* = 31) of couscous supplemented with 6% microalgae. Samples with (*) show a significant difference compared to the rest of the samples within the same sensory attribute.

The results of consumers’ intention to buy the newly formulated couscous are presented in Figure 6B. Twenty-three percent of the panel chose the statements “would usually buy” and “would always buy” for the Honey and the Smooth couscous, respectively. Regarding the Organic couscous, thirty-two percent of the participants would occasionally buy it. In the comments field of the sensory analysis sheet, the tasters referred to the Organic couscous as having an unpleasant fishy flavor, although it presented an appealing color. Overall, it can be concluded from the obtained results and the written and oral feedback that these innovative products will be greatly accepted in the market.

## 4. Conclusions

Innovative couscous products were successfully produced by adding algae biomass (6% *w*/*w*) to semolina flour. These products boast healthy properties imparted by the presence of functional biomolecules while maintaining the typical versatility of traditional couscous. Changes observed in the biochemical composition of the used biomass, due to different strain-specific characteristics and different culture conditions, led to couscous with different appearances and nutrient contents. The contents of protein (12% of the total energy), iron and magnesium (at least twice the RDV) were compatible with the provisions of Regulation (EC) No. 1924/2006, fulfilling the nutritional and health claims related to biocompounds. Additionally, the enrichment of couscous with algae permitted a higher protein in vitro digestibility and a clear improvement in the omega-ω6 to omega-ω3 fatty acid ratio, although it also increased the proportion of saturated fatty acids. Moreover, the innovative products exhibited acceptable cooking and sensory properties. Therefore, their position in the market of functional products is conceivably lucrative for food industries and algae biomass producers, which would, in turn, develop and commercialize functional couscous enriched in algae biomass.

## Figures and Tables

**Table 1:** Biochemical composition (% Dry weight (DW)) of the algae biomass and durum wheat semolina used in the experiments.

	Semolina	White *Chlorella*	Honey *Chlorella*	Smooth *Chlorella*	Organic *Chlorella*	Algaessence^®^
Protein% DW *	12	**40.9**	31.6	26.3	**56.6**	29.5
Carbohydrate% DW *	69	40.1	54.1	**58.1**	6.3	8.6
Lipid% DW *	2.4	**9.3**	7.2	7	8.5	4.4
Ash% DW *	0.9	5	4.1	4	10.2	18.3
Fiber% DW *	nd	nd	nd	nd	12.9	**33.4**
Chlorophyll mg/100 g **	0.2	**5.8**	8.9	89	**322**	123
Minerals mg/100 g **						
K	nd	376 ± 7.1	545 ± 13.5	700 ± 17.5	943 ± 23.5	1978 ± 30.8
P	nd	1191 ± 17.8	736 ± 13.0	1054 ± 30.0	2202 ± 42.6	886 ± 12.4
Mg	nd	61.9 ± 1.0	74.7 ± 2.2	109 ± 2.7	**229 ± 4.3**	**1366 ± 18.8**
Ca	nd	493 ± 3.3	210 ± 8.1	257 ± 6.5	**1119 ± 25.8**	**947 ± 9.0**
Fe	nd	7.1 ± 0.1	6.8 ± 0.5	10.2 ± 0.9	**167 ± 3.2**	**177 ± 4.1**
Cu	nd	0.6 ± 0.0	0.4 ± 0.0	0.4 ± 0.0	2.6 ± 0.0	1.6 ± 0.0
Mn	nd	4.8 ± 0.1	3.85 ± 0.0	4.03 ± 0.0	**11.74 ± 0.1**	**15.32 ± 0.1**
Zn	nd	14.5 ± 0.2	11.1 ± 0.2	16.2 ± 0.4	34.8 ± 0.5	19 ± 0.2

* Label information; ** determined in the current study; nd: not determined. Bold highlights the main differences in composition. DW: Dry weight.

**Table 2:** Color attributes (L*, a*, b*) of raw and cooked couscous with 6% algae addition. Control is couscous without algae addition.

	Raw Couscous			Cooked Couscous		
	L*	a*	b*	L*	a*	b*
Control couscous	74.88 ± 0.38 ^aA^	0.58 ± 0.05 ^aA^	36.18 ± 0.60 ^aA^	69.60 ± 1.64 ^aB^	−0.90 ± 0.29 ^aB^	28.62 ± 1.18 ^aB^
White couscous	65.75 ± 0.52 ^bA^	0.17 ± 0.05 ^aA^	33.73 ± 0.66 ^bA^	60.57 ± 1.41 ^bB^	−1.22 ± 0.14 ^aB^	29.58 ± 0.60 ^aB^
Honey couscous	65.75 ± 0.24 ^bA^	0.27 ± 0.07 ^aA^	50.59 ± 1.14 ^cA^	56.13 ± 1.64 ^cB^	−1.35 ± 0.41 ^aB^	44.30 ± 1.53 ^bB^
Smooth couscous	42.23 ± 1.02 ^cA^	−5.69 ± 0.43 ^bA^	29.11 ± 0.92 ^dA^	32.03 ± 0.61 ^dB^	−1.20 ± 0.13 ^aB^	15.17 ± 0.23 ^cB^
Organic couscous	28.64 ± 0.83 ^dA^	−10.40 ± 0.38 ^cA^	14.35 ± 0.73 ^eA^	18.97 ± 0.92 ^eB^	−2.99 ± 0.14 ^bB^	6.38 ± 0.34 ^dB^
Algaessence couscous	40.90 ± 0.28 ^cA^	−6.26 ± 0.09 ^bA^	21.56 ± 0.50 ^fA^	29.70 ± 0.26 ^dB^	−2.22 ± 0.13 ^cB^	11.33 ± 0.57 ^eB^

Means (*n* = 8) with different superscripts within the same column differ significantly (*p* < 0.05). Different capital letters following the same parameter (e.g., L* raw and cooked couscous) indicate that the difference in the means is significant at the 0.05 level.

**Table 3:** Power law parameters (α′, α″, b′ and b″) of couscous dough samples with algae addition.

	G′		G″	
	α′	b′	α″	b″
Control couscous	180,500 ± 33,374 ^a^	0.172 ± 0.005 ^a^	59,000 ± 12,348 ^a^	0.222 ± 0.000 ^a^
White couscous	418,700 ± 45,632 ^b^	0.175 ± 0.012 ^a^	147,800 ± 35,134 ^b^	0.228 ± 0.000 ^a^
Honey couscous	363,700 ± 47,640 ^b^	0.167 ± 0.012 ^a^	108,900 ± 15,463 ^a,b^	0.211 ± 0.000 ^a^
Smooth couscous	313,400 ± 16,590 ^b^	0.175 ± 0.007 ^a^	95,620 ± 6706 ^a,b^	0.209 ± 0.000 ^a^
Organic couscous	371,700 ± 50,500 ^b^	0.177 ± 0.001 ^a^	108,700 ± 16,527 ^a,b^	0.216 ± 0.000 ^a^
Algaessence couscous	387,100 ± 60,765 ^b^	0.183 ± 0.007 ^a^	118,900 ± 19,901 ^b^	0.198 ± 0.000 ^a^

The goodness of fit (R^2^) ranged from 0.906 to 0.997. Data shown are mean ± SD, *n* = 3. Means with different superscripts within the same column differ significantly (*p* < 0.05).

**Table 4:** Chemical profile and energy value of steam-cooked couscous with 6% algae incorporation. Control is couscous without algae addition. Data shown are mean ± SD, *n* = 3.

	Control Couscous	White Couscous	Honey Couscous	Smooth Couscous	Organic Couscous	Algaessence Couscous
Moisture (% *wt/wt*)	45.07 ± 0.13 ^d^	45.97 ± 0.14 ^c^	45.27 ± 0.12 ^c,d^	48.33 ± 0.66 ^a^	46.37 ± 0.42 ^b^	47.30 ± 0.11 ^b^
Protein (g/100 g)	6.58 ± 0.01 ^e^	**7.34 ± 0.02** ^b^	7.01 ± 0.08 ^c^	6.85 ± 0.04 ^d^	**7.51 ± 0.2** ^a^	6.72 ± 0.09 ^d,e^
Fat (g/100 g)	1.25 ± 0.21 ^c^	1.62 ± 0.00 ^a^	1.54 ± 0.11 ^a,b,c^	1.43 ± 0.06 ^a,b,c^	1.61 ± 0.10 ^a,b^	1.26 ± 0.19 ^b,c^
Ash (g/100 g)	0.71 ± 0.22 ^b^	1.00 ± 0.1 ^a,b,c^	1.37 ± 0.00 ^a^	0.84 ± 0.23 ^b,c^	1.20 ± 0.24 ^a,c^	1.39 ± 0.09 ^a^
Total carbohydrate * (g/100 g)	46.39 ± 0.41 ^a^	44.06 ± 0.10 ^c^	44.81 ± 0.17 ^b^	42.55 ± 0.28 ^e^	43.31 ± 0.33 ^d^	43.33 ± 0.16 ^d^
Energy value (kcal/100 g)	223.11 ± 0.64 ^a^	220.21 ± 0.40 ^b^	221.11 ± 0.55 ^b^	210.46 ± 0.64 ^c^	217.78 ± 0.46 ^d^	211.52 ± 1.28 ^c^
Minerals mg/100 g						
K (15% RDV ** = 300)	152.42 ± 5.18 ^a^	157.04 ± 2.87 ^a^	158.21 ± 10.10 ^a^	163.05 ± 8.75 ^a^	169.03 ± 10.72 ^a^	201.35 ± 12.55 ^b^
P (15% RDV = 105)	83.64 ± 2.98 ^c^	112.12 ± 4.03 ^a,b^	110.17 ± 13.03 ^b,c^	110.14 ± 7.97 ^b,c^	138.57 ± 18.08 ^a^	106.28 ± 6.99 ^b,c^
Mg (15% RDV = 56.2)	22.42 ± 0.96 ^b,c^	22.37 ± 0.66 ^c^	24.10 ± 4.03 ^b,c^	25.27 ± 1.50 ^b,c^	28.38 ± 1.55 ^b^	**64.20 ± 4.25** ^a^
Ca (15% RDV = 120)	10.46 ± 0.77 ^e^	24.10 ± 1.36 ^c^	15.39 ± 0.37 ^d,e^	16.43 ± 1.41 ^d^	**45.87 ± 2.41** ^a^	**39.26 ± 3.11** ^b^
Fe (15% RDV = 2.2)	1.25 ± 0.37 ^d^	1.08 ± 0.08 ^d^	5.05 ± 0.16 ^c^	1.37 ± 0.08 ^d^	**6.82 ± 0.33** ^a^	**5.74 ± 0.17** ^b^
Cu (15% RDV = 0.2)	1.57 ± 0.39 ^e^	2.73 ± 0.38 ^c,d^	5.31 ± 0.20 ^b^	1.78 ± 0.08 ^d,e^	7.06 ± 0.22 ^a^	6.44 ± 0.57 ^a^
Mn (15% RDV = 0.4)	1.40 ± 0.08 ^b^	1.79 ± 0.26 ^a,b^	1.76 ± 0.14 ^a,b^	1.68 ± 0.12 ^b^	2.17 ± 0.18 ^a^	1.50 ± 0.07 ^b^
Zn (15% RDV = 1.6)	0.36 ± 0.03 ^a^	0.37 ± 0.03 ^a^	0.46 ± 0.17 ^a^	0.36 ± 0.03 ^a^	0.42 ± 0.02 ^a^	0.39 ± 0.03 ^a^

Means with different superscripts within the same line differ significantly (*p* < 0.05). * Carbohydrate content was determined by difference. ** According to the recommended daily values (RDV) established by Regulation (European Community) No. 1924/2006; Directive No. 90/494 (EC). Bold highlights the main differences in composition.

**Table 5:** Main fatty acids in cooked couscous with 6% algae incorporation. Control is couscous without algae addition. Data shown are mean, *n* = 3.

ω-n	Fatty Acids (%)	Control Couscous	White Couscous	Honey Couscous	Smooth Couscous	Organic Couscous	Algaessence Couscous
	C14:0	0.1	0.1	0.2	0.1	0.2	0.8
	C15:0	0.1	0.1	0.1	0.1	0.2	0.1
	C16:0	18.8	21.7	21.1	20.8	19.7	20.1
ω9	C16:1c7	0.1	0.2	1.2	0.6	0.6	0.2
ω7	C16:1c9	0.1	0.2	0.2	0.2	0.5	0.5
ω6	C16:2	0.0	2.0	1.3	2.0	1.8	0.7
ω3	C16:3	0.0	0.2	0.3	0.7	0.0	0.7
	C17:0	0.1	0.3	0.2	0.2	0.6	0.3
	C18:0	1.8	1.9	2.2	1.5	1.7	1.6
ω9	C18:1c9	13.7	14.1	16.4	13.0	14.1	14.0
ω7	C18:1c11	0.8	0.7	0.7	0.7	1.0	1.0
ω6	C18:2	60.1	53.8	50.4	53.7	46.9	52.6
ω3	C18:3	3.3	3.2	3.5	4.6	9.0	5.6
	C20:0	0.2	0.2	0.2	0.2	0.2	0.2
ω7	C20:1	0.4	0.4	0.4	0.4	0.3	0.4
ω6	C20:2	0.0	0.0	0.0	0.0	0.0	0.1
ω6	C20:4	0.0	0.0	0.0	0.0	0.0	0.5
ω3	C20:5	0.0	0.0	0.0	0.0	0.0	0.1
	C21:0	0.1	0.1	0.1	0.1	0.1	0.0
	C22:0	0.2	0.2	0.2	0.2	0.2	0.3
	C24:0	0.2	0.2	0.2	0.2	0.2	0.2
Others		0.1	0.7	1.3	0.7	3.0	0.3
ƩSFA		21.5 ^a^	24.8 ^b^	24.4 ^c^	23.4 ^c^	23.0 ^d^	23.5 ^c,d^
ƩUSFA		78.4 ^a^	74.6 ^b,c^	74.3 ^b,c^	75.9 ^b,c^	74.0 ^b^	76.2 ^c^
ω3		3.3 ^a^	3.4 ^a^	3.8 ^b^	5.3 ^c^	9.0 ^d^	6.4 ^e^
ω6		60.1 ^a^	55.8 ^b^	51.7 ^c^	55.7 ^b^	48.7 ^d^	53.8 ^b,c^
ω6/ω3		18:1	17:1	14:1	11:1	5:1	8:1

Means (*n* = 3) with different superscripts within the same line differ significantly (*p* < 0.05). SFAs: saturated fatty acids; USFAs: unsaturated fatty acids; ω3: omega 3; ω6: omega 6.

## Data Availability

The datasets generated for this study are available on request to the corresponding author.
